# Recommendations for Integration of Chronic Disease Programs: Are Your Programs Linked?

**Published:** 2007-03-15

**Authors:** Amy B Slonim, Carol Callaghan, Lisa Daily, Barbara A Leonard, Fran C Wheeler, Charles W Gollmar, Walter F Young

**Affiliations:** Michigan Public Health Institute; Michigan Department of Community Health, Lansing, Mich; National Association of Chronic Disease Directors, Atlanta, Ga; National Association of Chronic Disease Directors, Atlanta, Ga; National Association of Chronic Disease Directors, Atlanta, Ga; National Association of Chronic Disease Directors, Atlanta, Ga; National Association of Chronic Disease Directors, Atlanta, Ga

## Introduction

Chronic disease programs in state public health agencies across the United States are increasingly taking action to integrate activities across single-disease program lines. The perceived benefits of program integration are the motivating force behind these actions, but there is little documentation about how to integrate programs, what the benefits are to program integration, and what barriers exist.

Public health agencies recognize benefits from chronic disease program integration because of the potential for efficient use of staff, funds, and surveillance and intervention efforts. Recent reductions in state and federal funding for chronic disease prevention have increased the need for partnerships to make achievement of program objectives possible, but there are constraints imposed by 1) funding that is specific to a disease or an organization (i.e., categorical) and may be unavailable to integrated programs; 2) barriers in the way agencies and organizations operate; and 3) program accountability that is not flexible enough to enable chronic disease integration. Despite these barriers, public health organizations see value in program integration, and there is a growing determination among public health professionals and policy makers to coordinate and link chronic disease public health programs.

Salinsky and Gursky discuss the role of public health in biodefense (1) and define program integration as *strategic alignment of resources for meaningful change*. Another definition of program integration is *state of combination or the process of combining into completeness and harmony* (2). Integration of chronic disease programs and the linking of resources can result in efficiency and improved communication and coordination among clients, providers, and government funding agencies (3). This essay describes guiding principles for successful chronic disease program integration initiatives and makes specific recommendations for chronic disease programs within state health agencies (SHAs); the National Association of Chronic Disease Directors (NACDD); and the National Center for Chronic Disease Prevention and Health Promotion (NCCDPHP), Centers for Disease Control and Prevention (CDC).

## Literature Review 

Transforming our public health system from one built on categorical programs to one integrated across programs presents challenges. Literature on integrated programs describes barriers to integration, discusses the risks of failed integration efforts, notes what the facilitating factors are that advance program integration, and provides recommendations for successful integration initiatives.

Lack of sufficient political will to make policy and organizational changes has been identified as a barrier for governments, education and research institutions, and professional associations that are addressing public health issues (4). Lack of leadership and organizational communication problems also have been identified as barriers to integration (1). Long-term program success is expected from collaborative integration initiatives, but there is a risk of destabilizing the organization and even organizational and communication chaos if integration initiatives are not managed adequately (5). 

Unpublished data from a survey of state chronic disease and tobacco control program managers identified a variety of barriers to program integration that included 1) categorical funding and accountability, 2) competition between organizations for funding (*turf protection*), and 3) concern that program identity, status, constituents, and focus on program outcomes or achievements may be lost (6). 

Factors that facilitated successful integration of early child health information systems were leadership, project governance, project administration, stakeholder involvement, organizational and technical strategies, technical support and coordination, financial support and management, and policy support and evaluation (7). An international program providing health assistance to developing countries identified important elements for effective and efficient health services as leadership, new partnerships, sectoral reform replacing a patchwork of categorical technical projects, and service integration that maps out a collaborative work program (8). 

State chronic disease programs have limited knowledge and many challenges to program integration but are moving toward improved program effectiveness and efficiency. This essay defines program integration, describes guiding principles for chronic disease program integration, and recommends actions for SHAs, NACDD, and CDC that will advance program integration.

### Definition of program integration

Clearly stating the purpose of a program integration initiative helps participants know what actions are being taken and why. Integration efforts should be recognized as a process that seeks consensus on long- and short-term goals and should not be considered an intended outcome.

We use the chronic disease program integration definition provided by Salinsky and Gursky (1) that integration is *the strategic alignment of chronic disease categorical program resources to increase the effectiveness and efficiency of each program in a partnership without compromising the integrity of categorical program objectives*. 

### Guiding principles of program integration

Certain basic guiding principles should be followed for chronic disease program integration to succeed. The principles and values offered here will help guide organizational planning and implementation of integration initiatives.

**Box 1. Principles of Program Integration**

- Do no harm to categorical program integrity.
- Clearly identify and state mutual benefits and opportunities.
- Be guided by efficiency-oriented processes.
- Be focused on health outcomes.
- Evaluate integration outputs and health outcomes.
- Engage stakeholders.
- Mobilize leaders.

Integration initiatives should maintain categorical program integrity by 1) respecting program identity; 2) preserving successful interventions and categorical program expertise; 3) helping sustain constituencies; 4) maintaining accountability to categorical program funding agencies; 5) acknowledging categorical program priorities, responsibilities, capacity, and successes; and 6) encouraging cooperation, coordination, and collaboration among program agencies. Integration efforts should not compromise categorical program goals.

Expected benefits to participating program partners should be clearly stated. All involved parties should  understand the benefits of the integration effort and how it will advance accomplishment of individual program goals. The planning process should identify and document mutual opportunities for integrative program development and policy advocacy work.

Program efficiency should be a primary objective of integration processes, and the effectiveness of categorical program partners should not be compromised. Efficient program integration actions should leverage human resources, use time and dollars wisely, avoid duplication of effort, and build on common program interests and objectives. These initiatives should minimize work added for program staff. The benefits of integration initiatives should be greater than the sum of individual program contributions.

Improved population health is the most important guiding principle for program integration. Integration activities should work toward improved health of common populations, address crosscutting issues (e.g., promotion of physical activity to prevent heart disease, diabetes, obesity), enhance the ability of programs to address diversity, reduce health disparities, be measurable, and use evidence-based or best-practice intervention methods.

Integration outcomes should be defined, monitored, and evaluated, and adjustments should be made to ensure continuous quality improvement of programs. The impact of individual and combined programs should be evaluated by using qualitative and quantitative methods when possible, and successful integration efforts should be publicly recognized by organizational leaders.

Successful integration initiatives engage key stakeholders, are meaningful and respectful of individual organizations, create and sustain strong internal and external partnerships, respect and value all perspectives, address needs and fears associated with integration, promote shared decision making, establish clear roles, share vision and accountability, and build internal capacity and staff competencies.

Integration initiatives require strong visionary leaders, a supportive organizational environment that guarantees dedicated resources and infrastructure, and communication strategies to reduce or remove barriers to the progress and effective oversight of institutional programs.

## Recommendations for Chronic Disease Program Agencies 

To stimulate chronic disease program integration initiatives in states, nationally, and for public health professionals, we suggest recommendations for three major public health sectors, SHAs, NACDD, and CDC, to help assure the success of chronic disease program integration initiatives.

### Recommendations for SHAs

Engage leadership at SHAs by 1) convening cross-program meetings, 2) defining the purposes of program integration, 3) identifying through collaboration linkage points across programs, 4) establishing and measuring program outcomes, 5) securing organizational endorsement of activities, and 6) keeping leaders informed about integration progress.

Develop crosscutting epidemiology and surveillance programs by 1) improving data collection and analysis, 2) integrating mapping of disease burden and risk factors, 3) supporting a multiprogram epidemiology workforce, 4) sharing technology resources, 5) packaging data reports to include multiple program areas, and 6) developing data sources for integrated public health information systems.

Leverage use of information technology by 1) collecting integration data, 2) assuring compatibility of administrative and management systems across programs and organizations, 3) sharing program information, and 4) developing collaborative work plans and population-based data repositories.

Build state and local partnerships by 1) strengthening professional relationships across programs, government and nongovernment organizations, and lay and professional groups; 2) striving for mutual benefits to individual programs; 3) including nontraditional partners; 4) establishing communication networks for staff and community partners; and 5) sharing information for mutual program development opportunities.

Develop integrated state plans by 1) establishing program integration as a normative and priority process; 2) guiding integration efforts; 3) making use of common and specialized data sources; 4) assuring that performance measures are identified, data are collected, and activities are realigned for integrated programming; and 5) convening internal and external partners regularly to monitor the integration progress.

Engage management and administration by 1) assessing readiness and organizational support for integration; 2) dedicating staff time to program integration; 3) conducting assessments to determine common linkages and program gaps; 4) planning and implementing strategies to engage partners, stakeholders, and program staff; 5) negotiating, tracking, and evaluating changes in financial management practices; 6) reorganizing as necessary to add or realign staff, budget, and program activities; and 7) engaging in joint problem solving among partners and stakeholders.

Implement integrated interventions by 1) developing interventions that identify specific targets for change, 2) assuring implementation of evidence-based strategies, 3) focusing on integration benefits and results, 4) identifying ways to share workload and resources, and 5) reviewing activities regularly for efficiency and effectiveness.

Evaluate integration initiatives by 1) determining the success of integrated program implementation, 2) measuring crosscutting performance outputs and outcomes, and 3) making programmatic adjustments based on evaluation findings.

**Box 2. Recommendations for State Health Agency Actions to Support Integration of Chronic Disease Programs**

- Engage SHA leadership.
- Develop crosscutting epidemiology and surveillance programs.
- Leverage the use of information technology.
- Build state and local partnerships.
- Develop integrated state plans.
- Engage management and administration.
- Implement integrated interventions.
- Evaluate integration initiatives.

### Recommendations for NACDD 

Many chronic disease prevention professionals responsible for implementation of program integration initiatives are members of NACDD. This national association of chronic disease professionals can play an important role in advancing program integration, and we make the following recommendations for NACDD to assist with chronic disease program integration.

Develop and disseminate tools by providing templates for integrated plans, promoting integrated program and surveillance models, and assisting with development of partner databases.

Provide educational opportunities by advancing program integration methods through workshops and program sessions at national chronic disease conferences and NACDD member teleconferences and meetings.

Provide outreach and recruit national partners by engaging national public health organizations focused on prevention and control of chronic diseases and related risk factors.

Reach beyond chronic disease programs by advocating program integration across state and federal categorical chronic disease programs and cooperating with state and federal agencies. 

Advocate leadership support by communicating with state and federal public health leaders and enlisting their support for chronic disease program integration. 

Assure continuity of integration initiatives by convening CDC and state program managers to encourage and advance planning, implementation, and evaluation of integration initiatives.

**Box 3. Recommendations for NACDD Action to Support Integration of Chronic Disease Programs.**

- Develop and disseminate program integration tools.
- Provide educational opportunities.
- Provide outreach and recruit national partners.
- Reach beyond chronic disease programs.
- Advocate leadership support.
- Assure continuity of integration initiatives.

### Recommendations for CDC 

CDC can play a significant role in the advancement of chronic disease program integration. The following recommendations for CDC to assist chronic disease program integration are offered with caution. National categorical programs must continue to set their goals, serve as effective program advocates, and provide technical assistance where needed.

Establish common terminology for improved communication with SHAs and categorical programs to guide program integration.

Modify state request for application (RFA) guidelines with wording that encourages program integration, provides flexibility, and creates an environment for SHAs to integrate programs creatively.

Develop performance standards and evaluation tools to assure development of quality program integration plans and universal evaluation tools that measure the value-added impact of program integration.

Build CDC staff capacity by implementing effective integration activities by establishing performance expectations, providing training, identifying performance incentives, and making program integration a part of institutional processes within NCCDPHP.

Develop integration tools by building integrated management information systems, crosscutting program evaluation tools, and integrated surveillance and evaluation plans and by documenting case studies about promising integration practices.

Allocate integration-specific resources by identifying 1) an internal program integration champion, 2) model integration strategies, 3) a clearinghouse for effective integration methods, and 4) communication channels among state and national partners to support program integration.

**Box 4. Recommendations for CDC Action to Support Integration of Chronic Disease Programs**

- Establish common language for SHAs and categorical disease programs.
- Review and modify RFA guidelines to encourage and make integration possible.
- Develop performance standards and evaluation tools appropriate to program integration.
- Build staff capacity.
- Develop integration tools.
- Designate integration-specific resources.

## Conclusion

Implementing the recommendations suggested in this essay should assist national- and state-level chronic disease programs achieve well-integrated programs. These recommendations can be summarized by the following actions: 1) engage the support of key partners and stakeholders, 2) educate and involve program staff and leaders about the value of integration activities, 3) plan for integration, 4) implement best practices and proven approaches, and 5) evaluate integration activities.

A strong commitment is needed from public health leaders and staff, and community partners if chronic disease program integration is to become a reality.
